# Can people living with a rare disease be independent? Inspiring personal stories

**DOI:** 10.1186/1750-1172-9-S1-O34

**Published:** 2014-11-11

**Authors:** Gabor Pogany

**Affiliations:** 1 Rare Diseases Hungary - Hungarian Federation of People with Rare and Congenital Diseases (HUFERDIS

## 

The Hungarian Williams Syndrome Association successfully organised the „Wing-Test” project supported by the Norwegian Fund and assisted by FRAMBU, the Resource Centre for Rare Disorders of Norway. In this presentation we first would like to present our innovative good practices from the perspective of the Participants and their families and summarize the results and effects on the life quality of these people and society.

What was this project about? The goal of the program was to prepare young adults (and their families) living with some kind of rare disease and disability to have their own independent life and work. Another goal was to form partnerships of strategic importance between the participants’ local services for better care. Therefore we organised two camps, one week each, as the first step to create the conditions of a long-lasting home. The target group of the project was 23 young people (14-35 years old) living with Williams syndrome or other similar disability, who are still living with their families, but they’ve already left school, or just having their last school years; and their families. During the one week program the youth with the help of the volunteers and the professionals could try themselves in different kind of work: gardening, forestry, tending to farm animals, housework, creative activities etc., while we were going to turn our attention to develop their abilities to become more independent. An important part of the program was to get in touch with local people, too.

